# *“If I have money, I cannot allow my baby to breastfeed only …”* barriers and facilitators to scale-up of peer counselling for exclusive breastfeeding in Uganda

**DOI:** 10.1186/s13006-020-00287-8

**Published:** 2020-05-15

**Authors:** Joseph Rujumba, Grace Ndeezi, Victoria Nankabirwa, Mary Kwagala, Michelle Mukochi, Abdoulaye Hama Diallo, Nicolas Meda, Ingunn M. S. Engebretsen, Thorkild Tylleskär, James Tumwine

**Affiliations:** 1 Department of Paediatrics and Child Health, College of Health Sciences, Makerere University, P.O. Box 7072, Kampala, Uganda; 2 School of Public Health Makerere University, Kampala, Uganda; 3 Department of Public Health, Faculty of Health Sciences, University of Ouagadougou, Ouagadougou, Burkina Faso; 4 Centre Muraz, Bobo-Dioulasso, Burkina Faso; 5 Centre for International Health, University of Bergen, Bergen, Norway

**Keywords:** Exclusive breastfeeding, Peer counselling, Support

## Abstract

**Background:**

Early initiation and exclusive breastfeeding for 6 months reduces infant morbidity and mortality and can positively impact on cognitive function. In Uganda, exclusive breastfeeding for 6 months is recommended but many women introduce alternative feeds early. Interventions to scale-up peer support provision for exclusive breastfeeding are limited. We explored the barriers, facilitators and solutions to scaling-up of peer counselling support for exclusive breastfeeding in Uganda.

**Methods:**

A qualitative study was conducted in Mbale District and Kampala City between April and July 2014. Data were collected through 15 key informant interviews with health workers and managers of organizations involved in child and maternal health as well as seven focus group discussions with peer counsellors who took part in the PROMISE EBF Trial (2006–2008), VHT members, mothers and fathers of children aged 1 year and below. Data were analysed using the content thematic approach.

**Results:**

The need for peer support for exclusive breastfeeding, especially for young and first-time mothers, was highlighted by most study participants. While mothers, mothers-in-law, friends and husbands were mentioned as major stakeholders regarding infant feeding, they were perceived to lack adequate information on breastfeeding. Health workers were mentioned as a key source of support, but their constraints of heavy workloads and lack of education materials on breastfeeding were highlighted. High community expectations of peer counsellors, the perceived inadequacy of breast milk, general acceptability of complimentary feeding, household food insecurity, heavy workload for women and unsupportive ‘work-places’ were key barriers to scaling-up of peer counselling support for breastfeeding. The peer counsellors who were part of the PROMISE EBF trial in Mbale, the village health team programme, health facilities, community groups, the media and professional associations emerged as potential facilitators that can aid the scaling-up of peer counselling support for breastfeeding.

**Conclusions:**

Peer support for breastfeeding is highly valued in this setting. The health system and health workers are regarded as the main facilitators to scaling-up of peer support for exclusive breastfeeding. Partnerships with village health teams (VHTs), community groups, role models, professional associations and the media are other potential facilitators to this scaling-up.

## Background

Exclusive breastfeeding is defined as the practice of giving only breast milk to the infant, without mixing with water, other liquids, herbal preparations or food in the first 6 months of life with the exception of vitamins, mineral supplements or medicines [1], has been identified as one of the key strategies for child survival [2, 3] Exclusive breastfeeding during the first 6 months provides all the required nutrients to the baby [2], offers protection against childhood infections, leads to better physical growth and neuro-development, and increases intelligence [4, 5]. In low and middle income countries, EBF in the first 6 months of life has been documented to have a protective effect against child mortality [5]. In high HIV prevalence countries, EBF is recommended for HIV-infected mothers in combination with appropriate antiretroviral medication [6]. Despite the clear benefits of exclusive breastfeeding, EBF rates in countries such as Uganda, are still sub-optimal [2]. The average EBF prevalence is 53.5% for the East African region of which Uganda is a part [7]. The 2016 national survey in Uganda revealed that 66% of children under 6 months are exclusively breastfed [2], lower than the national target of 80% [8] indicating the need for strengthening EBF promotion and support.

The practice of giving pre-lacteal feeds and early complimentary feeds is fuelled by a misconception that breast milk alone is not adequate for babies [9]. In addition, breastfeeding is still seen as a mother’s personal decision and consequently many mothers do not meet their own expectations of breastfeeding because of lack of support [10]. The social networks to which women belong can either be barriers or points of encouragement for breastfeeding [11, 12]. One major predictor of breastfeeding success is social support [13]. The woman’s family and other social support networks, which include the community, can be critical circles of support for breastfeeding mothers [14]. Founded on this circle of support is the community-based peer counselling (PC) program, designed to encourage and provide near home support to pregnant and breastfeeding women to practice exclusive breastfeeding (EBF) [15]. Desirable qualities of peer counsellors include being from the same neighbourhood, speaking the same language and sharing common cultural beliefs with their clients [16]. Peer counsellors can help the women make informed infant feeding choices and also provide breastfeeding information, emotional and practical support. A 2017 systematic review revealed that support to mothers increased the duration and exclusivity of breastfeeding [17]. Peer support programs also represent a cost-effective [18], individually tailored approach and culturally competent way to promote and support breastfeeding [19]. Nonetheless, interventions to scale-up peer support provision for EBF remain limited. Within the context of stagnation in EBF rates in Uganda [8], this qualitative study was conducted as part of the PROMISE Saving Brains, a follow-up study for the PROMISE EBF cluster randomised trial conducted in Uganda and Burkina Faso [20]. During the trial, peer counsellors were recruited and trained using materials based on the WHO courses on EBF. Peer counselors provided support to mother-infant pairs for exclusive breastfeeding. This study explored the barriers and facilitators to scaling-up peer counselling support for exclusive breastfeeding in Mbale and Kampala districts, Uganda, from the perspectives of peer counsellors, women, men and service providers. Understanding barriers and likely facilitators could provide insights on how to enhance and scale-up peer counselling support programmes for EBF promotion to optimise infant care.

## Study participants and methods

### Study design

A qualitative study was conducted between April and July 2014, as part of the Saving Brains Study in Uganda and Burkina Faso (PROMISE-SB) to explore the facilitators and barriers to scale-up of peer counselling support for EBF in Uganda. Qualitative research has the strength of describing the complexity of a phenomenon of concern in a natural setting [21]. It facilitates the understanding of social processes from the perspectives of the study participants, in this case mothers, fathers, peer counsellors, VHTs, health workers, community leaders and staff of organizations involved in maternal and child health care, informed by their lived experiences [21–23].

### Study sites

The study was conducted in Mbale District, Eastern Uganda and Kampala Capital City Authority (Central Uganda). Kampala Capital City Authority has a population of 1,507,000 people [24] and was included in the study for its unique status as a capital city and the presence of several service providers whose work has a bearing on promotion of EBF in Uganda. The city houses the Ministries of Health; Gender, Labour and Social Development, and is the headquarters of several organizations involved in the provision of maternal and child health services including EBF promotion. Mbale District has a population of 488,600 people with the majority (90%) residing in rural areas [24]. The district was purposively selected for inclusion in the study as the pioneer site where the Peer Counselling Support for EBF trial was implemented [20] and thus provided a unique opportunity to explore views of community members and volunteers who had been part of the PROMISE EBF cluster randomised trial in which mother-infant pairs in the intervention clusters received peer support for breastfeeding.

### Study participants

In Mbale district, the study participants included key stakeholders in relation to maternal and child health. These stakeholders were: district health team members, officials of faith based organizations, health workers, NGOs with community child health support programs, VHTs, peer counsellors who had participated in the provision of peer counselling support for EBF as part of the PROMISE EBF trial [20], and mothers and fathers of children less than 1 year old. Peer counsellors provided insights on their past, present and potential future roles, challenges and suggestions in relation to EBF support and promotion. Mothers and fathers of children less than one-year-old reflected on their feeding practices and the potential role peer counsellors could play in enhancing EBF.

In Kampala, study participants were purposively selected because they were involved in child health promotion including EBF. These included Officials from the Ministry of Health particularly those from the Child Health Division, health workers at Mulago National Referral Hospital, Baylor Uganda, The AIDS Support Organisation (TASO), Mamas Club, International Baby Food Action Network (IBFAN) Uganda, BRAC Uganda and the Uganda Paediatric Association [13].

## Data collection methods

### Focus group discussions

Focus group discussions (FGDs) of 6–8 members each were conducted to enlist collective views on barriers, facilitators and solutions to scale-up peer counselling support for exclusive breastfeeding in Uganda. Special interest was on exploring the potential for integration of peer counselling within the VHT strategy but also identifying other avenues for scaling-up peer counselling support for EBF. Overall, seven FGDs were conducted (one with peer counsellors, one with VHT members, four with mothers and one with fathers of children aged 1 year and below) in Mbale District. The FGDs were only conducted in Mbale – the district where the PROMISE EBF trial was conducted. Thus, it was possible to find peer counsellors and community member to engage in FGDs. The FGDs were conducted by two researchers, one as a facilitator and the other as a note taker. The discussions were conducted in *Lumasaba* and *Luganda*, the common languages spoken in the study setting. Discussions lasted for 45–60 min, were audio recorded and transcribed.

### Key informant interviews

Fifteen officials (eight from Mbale District and seven from Kampala) involved in the planning, implementation and monitoring of maternal and child health services, including infant feeding activities, participated in the study as key informants. In Mbale, key informants included selected community leaders where the PROMISE EBF trial was conducted, health workers and district health officials. In Kampala, informants included: one official each from Mulago National Referral Hospital, Ministry of Health Child Health Division, IBFAN, TASO Uganda, Mamas Club, Brac Uganda and the Uganda Paediatric Association.

An open-ended interview guide was used to collect data on the barriers, facilitators and solutions to scale-up peer counselling for exclusive breastfeeding in Uganda. One of the research team members conducted the key informant interviews together with a research assistant as a note taker. These interviews were audio recorded and transcribed by the research assistant and one of the research team checked the transcripts for accuracy.

The research team conducted daily de-brief meetings to share emerging issues from the study. At the end of the 7 FGDs and 15 key informant interviews no new information emerged regarding barriers and facilitators to scale up peer counselling for EBF, indicating that data saturation had been achieved.

## Data analysis

Preliminary data analysis occurred concurrently with the data collection. At the end of each day of data collection a research team meeting was held to share emerging issues and identify areas for further exploration. At the end of the data collection JR held a de-briefing meeting with co-researchers (GN and JKT) to share insights from the field. Further data analysis was conducted manually by JR using the content thematic approach, guided by the Graneheim and Lundman 2004 framework [25] to capture the latent and manifest content of the interview scripts. Study themes and sub-themes that emerged during data collection were refined following multiple readings of the interview transcripts. This process of continuous data analysis further assisted in the identification of themes and sub-themes as well as grouping of data accordingly. Verbatim quotations reflecting participants’ views on barriers and facilitators for the scale-up of peer support counselling for EBF promotion were identified and have been used in the presentation of the study findings.

## Ethical considerations

Ethical approval was obtained from Makerere University, School of Medicine College of Health Sciences, Research and Ethics Committee and Uganda National Council for Science and Technology. Permission was also obtained from management at each of the study institutions. Consent to participate in the study was obtained from all study participants. Research assistants were trained on the approach to data collection and the ethical issues of the study. At the end of data collection, JR de-briefed the members of the district health team on emerging issues. Results were presented at an annual Uganda Paediatric Association Conference.

## Results

### Characteristics of study participants

We conducted seven focus group discussions. Four for female and one for male parents of children less than 1 year, and two with community resource persons (Table 1). We interviewed 15 key informants (8 from Mbale and 7 from Kampala). Ten informants were female and five were male.

**Table 1:** Characteristics of study participants

Category of participants	Number of FGDs & KIIs	Study participants by gender
**Focus group discussions Mbale District**		
Peer counsellors	1	7 female
VHT	1	6 (3 female and 3 male)
Young mothers (18–24 years)	2	13 female
Older mothers (30–40 years)	2	17 female
Fathers (32–50 years)	1	7 male
**Total**	**7**	**50 (40 female &10 male)**
**Key informant interviews Mbale District**		
Community leaders	2	1 female & 1 male
District Health Team	2	1 female & 1 male
Non-Government Organizations	1	1 male
Faith based organization	1	1 female
**Health workers**	**2**	**2 female**
**Total**	**8**	**5 female & 3 male**
**Key informant interviews Kampala**		
Ministry of Health	1	1female
Mulago Hospital	1	1female
Non-Government Organizations	4	2 female & 2 male
Professional association	1	1 female
**Total**	**7**	**5 female & 2 male**

*FGD* Focus group discussion, *KII* Key informant interview

### Emerging themes for scale-up of peer counselling support for EBF

The results were grouped under 1) need for peer support; 2) barriers to scale-up peer support for EBF; and 3) facilitators for the scale-up of peer counselling support for EBF.

### Need for peer counselling support for exclusive breastfeeding

Most study participants in both the FGDs and key informant interviews mentioned that peer support for exclusive breastfeeding was needed by most mothers, but generally lacking. Peer support for EBF was especially needed for young mothers. Peer counsellors were highly valued and regarded as sources of accurate information on EBF, especially to help new and young mothers how to breastfeed and overcome the challenges they encountered. As one key informant observed:

*Peer supporters are needed especially for young mothers . . . some of them have not seen anyone breastfeed. They need guidance on how to position the baby on the breast but also for someone to answer their questions . . . thus peer counsellors, if trained, can help to address such concerns (KI Mbale District).*

Other study participants reasoned that some mothers often stop breastfeeding when they experience breast pain and such women need guidance:

*Some women stop breastfeeding when they experience pain in [their] breasts and such mothers need someone who has breastfed before to talk to and to encourage them… (FGD Peer counsellors, Mbale District).*

Evident in the above voices is that peer counselling support can help to motivate mothers to carry on with breastfeeding and address the challenges they face at home, especially during the early days of breastfeeding initiation.

Peer support for breastfeeding was also necessary to complement breastfeeding messages women receive at health facilities during antenatal care, maternity and postnatal care.

*Mothers are given some information on infant feeding during antenatal and delivery care. But most of the struggles with regard to breastfeeding manifest during the initial weeks after giving birth and at home; this is when peer counsellors can be of great help (KI Kampala).*

The above narrative reveals that while breastfeeding information is being introduced by health workers during antenatal and maternity care, mothers require follow-up support to address the practical challenges that emerge during the early breastfeeding period. Under such circumstances, peer counsellors are instrumental.

### Barriers to scale-up peer counselling support for EBF

Seven sub-themes emerged in relation to the barriers to scale-up peer counselling support for exclusive breastfeeding, including: limited resources, health facility challenges, HIV related challenges, cultural beliefs, economic barriers, lack of supportive policies and low male involvement (Table 2).

**Table 2:** Emerging themes on barriers to scale-up of peer counselling support for exclusive breastfeeding in Uganda

Organizing theme	Sub-theme
Limited resources and high community expectations	- Mobilization and support of peer counsellors - Motivation of peer counsellors - High community expectations
HIV related challenges	- Stigma and non-disclosure of HIV status - Confusing messages on HIV transmission through breastfeeding
Health facility challenges	- Few health workers - Lack of appropriate IEC materials on EBF promotion - Negative attitude of some health workers
Cultural beliefs and practices	- Breast milk is not enough especially for the boy child - Giving complimentary feeds is a norm, highly acceptable and widely practiced - Influence and advice from significant others e.g. mothers and mothers’ in-laws - Expressing breast milk a taboo and can kill
Economic barriers	- A belief that EBF only is for the poor who cannot afford supplementary feeds - Lack of food for the mother to produce adequate breast milk - Heavy domestic chores among women
Lack of supportive policies and programmes	- Work places not suitable for breastfeeding (lack space and short leave)
Low male involvement for maternal health	- Men do not attend antenatal clinics - Men are not informed and unsupportive of EBF

### Limited resources and high community expectations

Most study participants mentioned that scale-up of peer counsellors to support EBF was hindered by limited resources for training and remuneration of community resource persons. Peer counsellors also reported that often community members expected them to provide financial, medical and material support beyond the guidance and information they provided on EBF. These high expectations were attributed to community members being used to handouts, especially from politicians and program implementers working within a context of wide-spread poverty. As peer counsellors explained:

*Politicians give handouts whenever they come to meet community members. So, when we visit mothers to guide them on breastfeeding some of them expect us to have taken food, milk or to give them financial support when their children fall sick . . . Some of the mothers come to us asking for school fees for their children . . . (FGD Peer counsellors, Mbale District).*

The high community expectations were also echoed in all FGDs and by key informants, as one informant noted:

*These days, things have changed, even when you call community members for a brief meeting to educate them on something, they expect transport or a soda because politicians and some organizations are doing it. These expectations can make the peer support programme very expensive and unaffordable . . . (KI Mbale District).*

Some informants, especially those at the Ministry of Health and the District Health Team, mentioned that insufficient resources made it difficult to expand such initiatives and was a source of demotivation for community volunteers.

*Lack of financial incentives can be a challenge for peer counsellors. If there are no incentives, the peer support programme will have minimal impact like the village health team (VHT) programme which is good but most VHT members are not active except where there are partners which give them some incentives. Like VHTs, peer counsellors for exclusive breastfeeding cannot work fully as volunteers . . . they need facilitation such as bicycles, t-shirts and some allowance . . . (KI District Official, Mbale District).*

### The fear of stigma, HIV and breastfeeding challenges

Study participants mentioned that the fear of stigma and misunderstanding about EBF by women living with HIV were key barriers to peer support for EBF. It was noted that women living with HIV still fear and face stigma at home and in communities. Women who had not disclosed their HIV status, especially to their partners, were reported to fear being visited at home by peer counsellors in case it raised suspicion that they were HIV positive.

*HIV stigma is still a big problem. The current policy promotes exclusive breastfeeding for all women regardless of HIV status, but this message has not yet been well understood by both health workers and community members. So, providing peer support in such a setting may be a challenge (KI Kampala).*

The challenge of providing peer support for EBF in the context of HIV and breastfeeding, was further compounded by the rapid changes in information about breastfeeding and HIV which some of the mothers and peer counsellors found unclear and confusing.

*We have been told before that HIV positive women should not breastfeed, but recently we are being told that HIV positive women can breastfeed even for a long time without infecting their babies. These messages are really confusing us and [are] difficult to explain . . . (FGD Peer counsellors Mbale).*

In relation to the challenge of HIV and breastfeeding, VHT members added:

*There is fear of transmission of HIV from mothers to their children through breastfeeding among those who are HIV positive and it hinders the work of peer counsellors. Mothers don’t want to exclusively breastfeed for fear of infecting their children with HIV (FGD-VHTs Mbale).*

Similarly, most informants were concerned that the policies and guidelines on infant feeding continue to change, causing confusion among health workers as well as community members. Informants noted that it is difficult for the peer counsellors to convince HIV positive mothers to exclusively breastfeed.

*It has been known widely that HIV can be transmitted from mother to child through breastfeeding. These days breastfeeding is being promoted for all women, including HIV positive mothers, so communities have a lot of questions, and peer counsellors will encounter this confusion as a barrier in doing their work (KI Kampala).*

### Health facility challenges

Most key informants expressed concern that the health facilities were not well equipped to promote EBF and to recruit, train and support peer counsellors for EBF promotion. It was noted that health facilities are understaffed and lack appropriate information education and communication materials on EBF, making it difficult for them to train, support and supervise peer counsellors for the promotion of exclusive breastfeeding.

*Most health facilities lack posters and information on the promotion of breastfeeding. This is because most of the breastfeeding activities have been done under the programme for prevention of mother to child transmission of HIV, thus the focus has been on breastfeeding for HIV positive women as opposed to targeting the general women who are the majority. Besides, health workers are few and overworked. This makes it difficult for health workers to educate mothers and community members on breastfeeding (KI Kampala).*

Study participants also noted that some health workers do not have the right attitude to support EBF.

*The majority of health workers are not ready to support breastfeeding mothers. Health workers are not friendly and lack policies and guidelines to enforce and promote exclusive breastfeeding . . . (KI Kampala).*

Key informants also reported that most health workers lack adequate time in which to share detailed information with the mothers, especially during antenatal clinics and in the postnatal period, yet it would be a basis on which peer counsellors could build maternal knowledge.

*We know that, as health workers . . . we should promote breastfeeding, and peer counsellors can build on that information. But in practice we are very busy, so we do not give adequate information on breastfeeding to mothers . . . (KI Health Worker Mbale District).*

### Negative socio-cultural beliefs

Among the negative socio-cultural beliefs was the myth that breast milk is not enough especially for male infants and, as such, mixed feeding was deemed necessary to ensure satiety of the child, as noted by the women in a group discussion:

*Many women in our communities say that breast milk is not enough for the baby especially for boys. So, it is normal to give a child other feeds like porridge, soup, juice cow’s milk . . . (FGD Young women, Mbale District).*

In Mbale District expressing breast milk in case a mother was to be away was viewed as a taboo by most community level study participants who reasoned that expressing breast milk would lead to death of children and family isolation.

*Some community members believe that if a child who is not the one being breastfed drinks breast milk or uses a container in which breast milk was (stored); he/she would die, so people fear to express breast milk (KI Health Worker Mbale District).*

*Breast milk is only supposed to be in the mother’s breast but not ‘in home’ utensils. Who will ever eat food in your home if they discover you express breast milk? Tradition does not allow this (FGD Mothers Mbale District).*

In most FGDs men and women equated women who express breast milk to ‘milking them like cows’; a practice that was not acceptable. Women, especially those who had to stay away from home, were unable to exclusively breastfeed for the recommended time even with the peer counsellor available because of such beliefs.

Study participants also noted that the influence and advice from significant others such as mothers and mothers’ in-law, made the need for trained peer counsellors dispensable. In addition, it was noted that many people, including policy makers, view breastfeeding as natural and thus not requiring support.

*Most people take breastfeeding for granted, they think they know about it and others think breastfeeding is natural and does not require support which is not the case . . . (KI Kampala)*

Such beliefs serve to keep the role of peer counsellors unrecognized, thus limiting potential for scaling-up of the programme.

### Economic barriers

Some women of higher economic status were reported to prefer feeding their babies on breast milk substitutes rather than to exclusively breastfeed them. There was also a belief that exclusive breastfeeding was for those who were ‘poor’ and cannot afford complementary feeds.

*Breast milk alone is not enough for the baby. So, if I have the money . . . I cannot allow my baby to breast feed only. We buy porridge, milk, soda or juice . . . to help the child get satisfied (FGD Men Mbale District).*

Poverty was another barrier to limited activities and the scale-up of peer counselling support for EBF. Study participants identified the long work hours that women endured as a reason for their inability to exclusively breastfeed their children and the difficulties of being reached by peer counsellors. They also mentioned that because most mothers in Mbale were overworked and poorly fed, they would not produce adequate breast milk for their infants.

*Over working of women is a big challenge . . . they eat only once [a day] and maybe one type of food like cassava. The other crops we grow like beans and soybeans are sold to get money for school fees and other family needs. So, a woman who eats one meal a day and is overworked will not produce enough milk for the baby . . . (FGD men Mbale).*

### Lack of supportive policies and programs for EBF promotion

Lack of supportive exclusive breastfeeding promotion policies, incentives in ‘workplaces’ including a lack of baby-friendly spaces, designated breastfeeding areas and short maternity leave (only 3 months) were cited as some of the barriers to EBF by women in the formal employment sector.

*Most working mothers are given 90 working days for maternity leave but due to financial constraints even those days are not fully given to the baby since the mother has to run here and there looking for survival and trying to make ends meet (KI Kampala).*

Participants also noted a lack of clear policies and guidelines on the selection, training and support of peer counsellors for EBF as a major barrier.

Women in the informal sector were at a seemingly greater disadvantage given that they rely on daily earnings and are unable to take leave from work to exclusively breastfeed their children. They also lacked space at the workplace for breastfeeding and for their children to stay with them. As some women explained:

*If a mother can support herself or if she has a daily income so that she doesn’t leave home, she can be able to stay home with her baby and breastfeed. But now some of us are casual laborers. We go to pick coffee at BCU and there is nowhere you can place your baby or even breast feed . . . so it becomes a challenge (FGD-mothers, Mbale District).*

### Low male involvement in child health

The low levels of male involvement and support for maternal and child health programs like antenatal care, was listed as a barrier to the scale-up of peer support for EBF. It was noted that owing to their lack of involvement in decision making on maternal and child health, most men were uninformed and yet were predominantly the decision makers in the homes.

*In most cases husbands rarely interface with the health system. Many do not attend antenatal care with their wives, thus missing out on health education including on infant feeding but are influential on how to feed their children. So, they rely on informal sources of information such as their mothers and sisters who may not have accurate information on EBF . . . (KI Kampala).*

*Men are important decision makers and can hinder exclusive breastfeeding if not brought on board. For example, it can be difficult for a woman to refuse to give milk or any other feeds that a man has bought to the child (KI Kampala).*

In such settings, it becomes difficult for peer counsellors to access women and support them on EBF if their partners are uninformed and not involved.

### Facilitators to scale-up peer counselling for EBF

Several facilitators identified mainly structures which could be used to aid scaling-up of peer counselling support for exclusive breastfeeding include, health facilities, VHTs, the media, role models, community and faith-based groups as well as professional associations (Table 3).

**Table 3:** Thematic presentation of facilitators and solutions to scale-up peer counselling support for exclusive breastfeeding in Uganda

Structure	Solution
Health facilities	- Integrate breastfeeding messages in health education, antenatal, maternity, postnatal and outreach services - Train and supervise peer counsellors
Village heath teams	- Ministry of Health Structure, available in most districts - Already involved in maternal and child health
The media (radio and newspapers)	- Dissemination of information on breastfeeding - Opportunity to correct misconceptions
Role models	- Use of influential and respected community members to promote EBF
Existing peer counsellors in Mbale	- Knowledgeable and can provide opportunity for learning
Community and faith-based groups	- Breastfeeding promotion in existing membership of women/mothers - Widespread and trusted
Professional associations	- Uganda Paediatric Association, Nurses and Midwives Association with country-wide reach - Technical expertise spread throughout the country

### The health system to promote peer support for EBF

The existent health system was identified as a major facilitator to the scale-up of peer counselling support for exclusive breastfeeding in Uganda. This scale-up was dependent upon the countrywide existence of health facilities at different levels staffed by knowledgeable health workers who can train, supervise and mentor peer counsellors to support mothers to exclusively breastfeed. Most study participants noted that health workers can provide updated information on breastfeeding and be the points of call for peer counsellors whenever they require more information.

VHTs, a Ministry of Health community-based structure consisting of volunteers geared at addressing community health needs, was identified as another critical element of the health system to facilitate the scale-up of the peer counselling support for breastfeeding. Study participants noted that because of the presence of the VHTs in most districts of Uganda, incorporating peer counselling for EBF in VHT training and activities would ensure wider coverage and reach more women with such support.

Discussions with VHT members in Mbale District revealed that their current work and training did not include much information on breastfeeding. In addition, VHTs lacked appropriate information education and communication materials on breastfeeding to guide community mobilization, education and support activities. The health system thus presents an opportunity to ensure that exclusive breastfeeding is promoted both at the community and health facility levels.

### Use of existing peer counsellors in Mbale and support groups

The study findings revealed that existing peer counsellors in Mbale District initiated as part of the PROMISE EBF trial, were another facilitator for the scale-up of the peer counselling programme. It was noted that these peer counsellors aided learning for others through exchange visits.

*For us, we have been providing peer support to mothers. Even when the study ended, we still provided information and guidance to mothers near us . . . we can share this experience with other peer counsellors (FGD Peer counsellors, Mbale District).*

Existing community and faith-based groups like women’s groups, were also identified as another facilitator for the scale-up of peer support for EBF. Study participants noted that church, savings and credit groups, and women and famers groups can disseminate information about the benefits of EBF and promote it among group members. Also, group leaders could be trained as peer counsellors to provide the needed support to breastfeeding women within their groups and communities.

### Use of role models

Use of role models who are influential and respected members of society to promote breastfeeding was another possible facilitator for EBF support mentioned by both key informants and focus group discussion participants. It was noted that engaging models such as the speaker of parliament, the first lady, musicians and cultural leaders can help to promote breastfeeding and enhance the work of peer counsellors. Most study participants stressed the need to provide accurate information to such community role models to ease their mobilization and community education.

*Reaching out to church leaders to provide them with information on breastfeeding can help them to integrate such messages in teaching sermons. For example, on March 25*^*th*^*every year we celebrate Mary’s day, so we could add breastfeeding messages on the theme for such celebrations to popularize breastfeeding and ask people to volunteer as peer counsellors . . . (KI Mbale District).*

In relation to role models, other informants explained:

*Role models such as the first lady or the speaker of parliament who are women themselves, if given appropriate information on breastfeeding . . . can be good champions for its promotion. I heard on news that the speaker of parliament has initiated a breastfeeding corner in parliament . . . (KI Kampala)*

*Some of the role models such as musicians, religious, political and cultural leaders are well known, respected and people believe in what they say. Such people should be trained and given summary information on the benefits of breastfeeding, challenges mothers face and how they can be addressed . . . (KI Kampala)*

What emerges from the above narratives is that role models can leverage their status and popularity to promote peer support for EBF but require up-to-date information.

### Use of the media

The use of the media, both electronic and print media, also emerged as a potential facilitator for the scale-up of peer counselling support for exclusive breastfeeding. It was noted that the media, particularly local radio stations, were good avenues to educate community members about breastfeeding and the peer counsellors programme for the support of EBF. The media was also seen as an opportunity to counter misconceptions about exclusive breastfeeding.

### Professional associations

Engaging professional associations such as the Uganda Paediatric Association, Nurses and Midwife associations was also identified as a facilitator for the promotion and support of peer counselling for EBF. This suggestion was premised on having countryside technical membership who can assist in the training, supervision and mentorship of peer counsellors in Uganda.

## Discussion

We explored the barriers and facilitators to scale-up peer counselling support for exclusive breastfeeding in Mbale and Kampala Districts, Uganda. The findings revealed that peer support for women to practice exclusive breastfeeding is highly needed, especially for young mothers. This need was linked to declining opportunities within communities for young mothers to learn about breastfeeding. The need for peer counsellors to support exclusive breastfeeding was also documented in earlier Ugandan studies [20, 26] and other settings [27]. These findings taken together stress the need to prioritise scaling-up of peer counselling support for EBF in Uganda and other low-income settings. Support to mothers has been associated with increased duration and exclusivity of breastfeeding [17].

Our study established that scaling-up of peer counselling support for EBF is hindered by high community expectations from peer counsellors in the form of financial and material support. The high community expectations documented in our study reflect the growing challenge of incentivising community volunteers in a highly monetised setting. This finding shows that plans for scaling-up peer counsellors for EBF need to go hand in hand with clear plans and provision for their motivation. It is indeed important that the needs of community volunteers are met owing to the amount of work that they do [28]. Limited resources translated into difficulties in identification, training, supervision and motivation of a critical mass of peer counsellors to effectively provide the needed support for EBF on a sustainable basis. Consistent with our findings, inadequate resources have been documented to hinder scaling up of breastfeeding promotion in low and middle income countries [29].

The belief that women living with HIV should not breastfeed for fear of infecting children was dominant in the study setting. This could be explained by previous information disseminated to the public on the risk of HIV transmission through breastfeeding. In another study in Kampala, the fear of HIV infection from mothers to their babies as a result of breastfeeding was highlighted as a barrier to exclusive breastfeeding promotion [30]. It is important to note that whereas under the current option B plus guidelines being implemented in Uganda since 2012, exclusive breastfeeding for the first 6 months is promoted for HIV negative and positive women. Our findings depict persistent knowledge gaps in understanding the role of antiretroviral treatment in making breastfeeding safer for HIV exposed babies. These findings highlight a need to intensify communication about guideline changes so that peer counsellors can aid this endeavour.

Other barriers to scaling-up of peer counselling support for EBF were related to health system challenges. Most health workers were overburdened and unable to effectively support the training and mentoring of peer counsellors for EBF. Health workers also lacked information education and communication materials on EBF. These gaps mean that health workers are not well equipped to create an enabling environment in which peer counsellors are expected to promote EBF. Similar barriers have been highlighted to hinder scaling-up of peer counsellors for EBF elsewhere [29]. Health workers’ lack of time to access adequate information about breastfeeding practices especially EBF, has been widely documented in the literature [31, 32] again indicating how it can hinder the scaling-up of peer counselling for EBF.

Negative cultural beliefs were a key barrier to peer support for EBF promotion. Such beliefs included: ‘that breast milk is inadequate, especially for male infants’; ‘expressing breast milk as a taboo’; and the negative influence of significant others. Cultural beliefs surrounding infant feeding choices such the a belief that women are not able to produce enough milk for their babies have been documented elsewhere [29, 33]. The widespread acceptability of complimentary feeding and the role of family members as sources of influence have also been documented elsewhere [31]. The implication here is that scale-up of peer counselling support for EBF should go hand in hand with addressing such widespread negative cultural beliefs on infant feeding and addressing negative influences from family and other sources of information.

Our study revealed that economic barriers such as lack of food and heavy domestic chores make it difficult for mothers to produce adequate breast milk to exclusively breastfeed their babies for 6 months. This finding is not surprising given that 25% of Ugandans are estimated to be living below the poverty line [34]. Besides, in many Ugandan societies, women are overburdened by care work which is likely to lead to stress and affect breast milk production. While most community members believed that exclusive breastfeeding was pro-poor, some community members perceived exclusive breastfeeding to be for mothers who could not afford alternative feeds and a likely barrier to exclusive breastfeeding promotion.

Other barriers to the scale-up of peer support for EBF were lack of supportive policies especially for working women both in the formal and informal sectors, and low male involvement in maternal and child health programmes. It was noted that scale-up of peer counselling for EBF requires a supportive environment at places of work and at home. The implications for EBF of long working hours on mothers and unfavourable workplace policies like short maternity leave was consistent with the findings of a study in Nigeria where such policies were cited as barriers to EBF [35].

With regard to facilitators for the scaling-up of peer counsellors for EBF, our findings revealed that the existing health system can aid this endeavour. It was noted that exclusive breastfeeding messages in antenatal and postnatal care clinics as well as community outreach sessions can be strengthened, and peer counsellors can build on them. In addition, health workers can play a critical role in training and supervision of peer counsellors for EBF. The VHTs which are part of the Ugandan Ministry of Health system were also identified as a structure which can facilitate scaling-up of peer counsellors for EBF. One way is to integrate peer counselling for EBF in the training of VHTs. Indeed, in Mbale District, some of the peer counsellors who were initiated under the PROMISE EBF study were already part of the VHT programme and were promoting EBF. The need to integrate peer counselling and EBF promotion in existing programmes has been identified as an enabling factor for EBF [29].

Partnerships with the media for information dissemination and correction of EBF related misconceptions were another key facilitator identified for scaling-up of peer counselling for EBF. Other facilitators identified included: use of existing peer educators for further learning; use of role models, engaging community and faith-based groups as well as professional associations such as the Uganda Paediatric Association and Uganda Nurses and Midwives Associations. These organisations were seen as avenues for community mobilisation to identify and train peer counsellors for EBF. Building a coalition with various stakeholders including opinion leaders, community groups, civil society organisations has been identified as a prerequisite for eliciting commitment for scaling-up peer counselling support for EBF [29].

The findings of our study should be interpreted in light of the following limitations. 1) given the qualitative nature of our study we were unable to provide quantifiable results on barriers and facilitators to scaling-up of peer counselling support for EBF. 2) Since the Peer Support Programme for EBF is not yet being implemented in many settings in Uganda, most of our study participants highlighted what they thought could hinder scale-up, as opposed to actual hindrances. However, the views from peer counsellors and Key Informants from Mbale District where the programme had been implemented helped to overcome this limitation. Future studies should be considered once the peer support programme has been implemented on a number of sites.

## Conclusion

Peer support for breastfeeding is a highly valued intervention in the study setting. The health system and health workers are regarded as the main facilitators to scaling-up of peer support for exclusive breastfeeding. This could be achieved by using the existing VHTs. Partnerships with existing women and community groups, role models, professional associations and the media are other potential facilitators. There is need to emphasize the benefits of EBF across all income groups and lobby for more favourable policies in support of the practice. Provision of updated information and education materials, health facilities and community leaders would be an avenue through which the information can be disseminated.

## Data Availability

The datasets generated during and/or analyzed during the current study are not publicly available due to ethical reasons but are available from the corresponding author on reasonable request.

## Abbreviations

EBF: Exclusive Breastfeeding
FGDs: Focus group discussions
NGOs: Non-Government Organisations
UPA: Uganda and the Uganda Paediatric Association
VHTs: Village Health Teams
WHO: World Health Organization
